# Global knockout of VEGFB improves lipoprotein lipase activity leading to an improved lipid profile during diabetes

**DOI:** 10.3389/fphar.2026.1759414

**Published:** 2026-02-18

**Authors:** Hualin Wang, Rui Shang, Chae Syng Lee, Bahira Hussein, Brian Rodrigues

**Affiliations:** Faculty of Pharmaceutical Sciences, University of British Columbia, Vancouver, BC, Canada

**Keywords:** diabetic cardiomyopathy, fatty acid metabolism, LPL, NEFA, VEGFB

## Abstract

Diabetes affects over half a billion people worldwide, with cardiovascular disease being its leading cause of death, either occurring secondary to atherosclerosis or due to an intrinsic defect in heart muscle (diabetic cardiomyopathy, DbCM). One instigator for DbCM is impaired cardiac metabolism characterized by excessive fatty acid (FA) delivery and utilization by the heart, causing oxidative stress and toxic lipid accumulation. Inhibition of vascular endothelial growth factor B (VEGFB) has been shown to counter these factors associated with abnormal cardiac metabolism by inducing metabolic flexibility and preventing cardiac lipid accumulation in Type 2 diabetes. However, its impact on lipoprotein lipase (LPL) and the sources of FA for cardiac use in Type 1 diabetes is unknown. Global *Vegfb* knockout (Vegfb^KO^) in rats caused limited phenotype and cardiac transcriptome changes under normal conditions but notably reduced cardiac LPL activity, probably by impeding LPL translocation from cardiomyocyte to the coronary vasculature. Under streptozotocin (STZ)-induced diabetes, Vegfb^KO^ rats exhibited increased cardiac LPL activity, protecting animals from dyslipidemia, decreased plasma saturated FA, and provided a safer cardiac FA source, LPL-derived FA. Knockout of *Vegfb* also protected animals from DbCM by inhibiting excess FA oxidation, preserving angiogenesis and alleviating cell death in the heart. Inhibiting VEGFB may offer a promising therapeutic approach to address the current lack of mechanism-based treatments for DbCM.

## 1 Introduction

The heart is a high energy-demand organ which utilizes multiple energy sources, primarily glucose (30%) and FA (70%), with lactate, ketones, and branched-chain amino acids playing minor roles under physiological conditions (Lopaschuk et al., 2010; Ritterhoff and Tian, 2023). Related to FA, this substrate is obtained through lipolysis of circulating triglyceride (TG)-rich lipoproteins by cardiac LPL, but also through adipose tissue lipolysis and cardiac intracellular TG breakdown (An and Rodrigues, 2006; Kim et al., 2012). Of these, LPL-mediated action on lipoproteins is suggested to be the main source of FA for cardiac use (An and Rodrigues, 2006).

LPL is expressed in multiple organs, including the heart, skeletal muscle, and adipose tissue, with the heart having the highest expression of this enzyme. Interestingly, although lipoprotein-TG breakdown by LPL occurs at the vascular endothelial cell (EC) lumen (Chiu et al., 2016; Lee et al., 2023), ECs cannot produce LPL. Instead, within the heart, LPL is produced by cardiomyocytes. After secretion from these cells, LPL binds to heparan sulphate proteoglycans (HSPG) on the cardiomyocyte surface. Subsequently, endothelial heparanase releases LPL from HSPG, allowing it to cross the interstitial space and bind to glycosylphosphatidylinositol-anchored high-density lipoprotein binding protein 1 (GPIHBP1) at the basolateral side of ECs. GPIHBP1 helps LPL across ECs to the vascular lumen. At this location, LPL hydrolyzes lipoprotein-TG to FA with GPIHBP1 also serving as a platform for this lipolysis (Chiu et al., 2016). In summary, the heart uses LPL to regulate the precise amount of FA delivered for energy utilization. It should be noted that under fasting or stress conditions such as diabetes, the contribution of FA towards cardiac metabolism increases due to AMPK activation (Shang et al., 2024; Augustus et al., 2003) or impaired insulin signaling (An and Rodrigues, 2006; Lopaschuk et al., 2010). The contribution of NEFA also increases due to augmented adipose tissue lipolysis (Ballard et al., 1960; Avtanski et al., 2024; Shang et al., 2024). In an energy deprivation state or diabetes, AMPK signalling is turned on, which will upregulate CD36 levels in the heart (Augustus et al., 2003), the key transporter for NEFA uptake (Coburn et al., 2000; Hwang et al., 1998; Bharadwaj et al., 2010; He et al., 2018). AMPK also increases the translocation of LPL from cardiomyocyte to the endothelial lumen surface. However, when excessive FAs are provided to the heart, this may itself modulate coronary LPL activity in multiple ways, including: a) displacing LPL from EC surface binding sites for degradation in the liver, b) directly inactivating LPL enzyme activity, and c) impairing LPL vesicular trafficking to the myocyte surface through the caspase-3-mediated cleavage of PKD (Bengtsson, G., et al. 1980; Wang et al., 2009, Kim et al., 2009, Kim et al., 2008b).

As glucose uptake and utilization are impaired following diabetes, the heart is obliged to use FA almost exclusively (Lopaschuk et al., 2010; An and Rodrigues, 2006). Multiple mechanisms operate to make this achievable, including augmented adipose tissue lipolysis with elevated plasma non-esterified FA (NEFA). Under these conditions, NEFA utilization exceeds LPL-derived FA (Yu et al., 2005; Wang et al., 2011; Kim et al., 2009; Wang et al., 2009; Puri et al., 2019) such that the heart loses its ability to choose its preferred FA source. A downside to the increased NEFA supply, added to the limited oxidative capacity due to diabetic microangiopathy (Laakso, 2011; Hinkel et al., 2017; Adameova and Dhalla, 2014), is the accumulation of toxic lipids in the heart, which has been reported to decrease insulin sensitivity and correlate with cardiac dysfunction (Blanchette-Mackie et al., 1989; Shang et al., 2021). Additionally, augmented FA oxidation increases ROS, which paired with the lipid metabolite accumulation, could be potentially detrimental, leading to cell death (Borradaile and Schaffer, 2005; Wilson et al., 2018; Volpe et al., 2018; van de Weijer et al., 2011; Wende et al., 2012; Kim et al., 2012). In this environment, protection against excessive utilization of NEFA and restoring metabolic flexibility in the diabetic heart would be advantageous. Targeting VEGFB has been shown to influence all the above approaches (Falkevall et al., 2023; Hagberg et al., 2010; Falkevall et al., 2017; Hagberg et al., 2012), and may contribute to the prevention of diabetic heart disease.

VEGFB belongs to the vascular endothelial growth factor (VEGF) family, and is mainly expressed in highly metabolically active organs like the heart, skeletal muscle and adipose tissue (Kivela et al., 2014). As the expression of *Vegfb* and mitochondrial genes and proteins are coordinately regulated (Bry et al., 2010), VEGFB plays an important role in metabolism. In this regard, *in vitro* studies demonstrate that VEGFB can augment FA transporters and decrease glucose utilization (Hagberg et al., 2010; Moessinger et al., 2020). Interestingly, VEGFB can indirectly induce angiogenesis by increasing the binding of VEGFA to VEGFR2 (Karpanen et al., 2008; Mould et al., 2005; Zhang et al., 2009; Robciuc et al., 2016; Mallick et al., 2022; Holmes and Zachary, 2005; Kivela et al., 2014). Although VEGFB treatment is thought to be largely beneficial (Arjunan et al., 2018; Bry et al., 2010; Huusko et al., 2012; Kivela et al., 2014; Rasanen et al., 2016; Shang et al., 2021; Shang et al., 2024), especially in myocardial infarction and ischemia reperfusion, there is also evidence indicating that inhibiting VEGFB is not necessarily harmful. Thus, Vegfb^KO^ mice or rats are viable and fertile, with smaller heart sizes but normal cardiac vasculature and heart function (Kivela et al., 2014; Bellomo et al., 2000). A similar effect on blood vessels is also observed in adipose tissue-specific *Vegfb* knockdown mice (Falkevall et al., 2023). Intriguingly, although *Vegfb* knockout has no observable metabolic phenotype under physiological conditions, the situation is reversed when these animals are challenged (Dijkstra et al., 2014; Kivela et al., 2014; Falkevall et al., 2023). For example, in Type 2 diabetes (T2D) generated using a high-fat diet (HFD) (Falkevall et al., 2023; Kivela et al., 2014; Ning et al., 2020; Hagberg et al., 2012; Luo et al., 2022) or in *db/db* mice (Hagberg et al., 2012; Falkevall et al., 2017), Vegfb^KO^ animals demonstrated improved metabolic flexibility and protection of non-adipose organs from lipotoxicity.

Currently, the beneficial effects of VEGFB inhibition are predominantly observed in mouse models of T2D. Whether *Vegfb* knockout would be cardioprotective in T1D is currently unknown. Our data suggest that inhibition of VEGFB will help defend the myocardium against diabetes-induced susceptibility to cardiomyopathy.

## 2 Materials and methods

### 2.1 Experimental animals

Our animal protocols were approved by the University of British Columbia Animal Committee (A21-0052 and A22-0014). All animal procedures performed conform to guidelines and regulations published by the Canadian Council on Animal Care. In this study, all animals had free access to food (LabDiet PicoLab Rodent 20 No. 5053) and reverse osmosis water and were housed in standard cages with 12 h of light/dark cycles. Vegfb^KO^ and Wildtype (WT) littermate male rats 7-12 weeks old were used for various studies. Rats were euthanized using a single dose of 100 mg/kg i.p. injection of sodium pentobarbital (Euthanyl). Thoracotomy was performed for organ collection when the toe pinch and corneal reflexes were absent.

Vegfb^KO^ rats on the Sprague Dawley background were generated with zinc-finger nuclease technology and kindly provided to us by Dr. Alitalo (Kivela et al., 2014). Beta-glucosidase gene (*lacZ*) was inserted into the exon 1 region of *Vegfb* to disrupt the gene. PCR was run on ear notch samples with the primers 5′-CCT​GCT​CCG​TCG​CTT​GCT​G-3′, 5′GGT​CTG​CTT​TCT​GAC​AAA​CTC​G-3’ (Vegfb F,R) and 5′-GGGGCCATCAAACTGGGAC-3′(lacZ R) to check the genotype.

### 2.2 Streptozotocin (STZ)-induced diabetes

The selective beta-cell toxin STZ (Sigma) is used to lower insulin and generate a rat model of poorly controlled T1D (Rodrigues et al., 1997; Wang et al., 2011). Following intravenous administration of STZ (55 mg/kg; D55), animals became hyperglycemic and hypoinsulinemic within 24 h and were monitored for 4 days. D55 animals mimic insufficient glycemic management in T1D patients where multiple finger pricks and daily insulin injections could mean poor patient compliance and repeated exposure to bouts of hyperglycemia. Given the sex differences in the susceptibility to STZ diabetes (Goyal et al., 1987), only male rats were used for this study.

### 2.3 LPL activity assay

LPL activity was performed as previously described (Shang et al., 2021; Shang et al., 2024). Briefly, the heart was retrogradely perfused with Ca^2+^-free Krebs-Ringer HEPES buffer to clear blood from the coronary vasculature. Subsequently, buffer was switched to fresh Ca^2+^-free Krebs-Ringer HEPES buffer containing 5 U/mL heparin (Sandoz) to displace LPL from the coronary vasculature at a flow rate of 8 mL/min. The coronary perfusates were collected for 15 s over 10 min. LPL activity was measured by the hydrolysis of a [^3^H]triolein (Perkin-Elmer) substrate emulsion. The perfusate samples mixed with the reaction mix (piperazine-N,N′-bis(2-ethanesulfonic acid), albumin, MgCl_2_ and chicken serum) were incubated at 30 °C for 30 min. Sodium [^3^H] oleate was extracted via centrifugation at 4 °C and 3500 rpm for 30 min, and measured by liquid scintillation counting.

### 2.4 Pancreatic insulin extraction

Pancreatic insulin was extracted by the acid-ethanol method. Briefly, frozen pancreas tissue was ground to a powder then homogenized and incubated in Acid-Ethanol (1.5% HCl in 70% ethanol) solution overnight at −20 °C. Aqueous layer was obtained after centrifuging at 2000 rpm for 15 min, followed by neutralizing Acid-Ethanol aqueous extract with 1M Tris (pH 7.5) in 1:1 ratio. Extracted pancreatic insulin was determined using an insulin ELISA kit (ALPCO). The pancreatic insulin concentration was normalized by the protein concentration of neutralized insulin extraction.

### 2.5 Metabolic analysis

Tail vein blood glucose was measured by a glucometer (AccuSoft) and glucose test strips (Accu-Chek Advantage). Blood was collected from the thoracic cavity by an EDTA-coated tube (Sigma) and centrifuged (1500 rpm at 4 °C for 15 min) to collect plasma. Plasma NEFA (Abcam) and TG (Stanbio) were measured by commercial kits using the manufacturer’s protocols. Plasma insulin was detected by the insulin ELISA kit (Alpco). Plasma and cardiac FAs and TG were analyzed by high-performance liquid chromatography-mass spectrometry (HPLC-MS; Waters 2690 Alliance HPLC, Milford, MA) as previously described (Puri et al., 2019; Shang et al., 2021). Briefly, plasma and cardiac lipids were extracted and solubilized by the modified Folch method with chloroform:methanol:acetone:hexane (4:6:1:1 v/v/v/v). The chromatographic separation of TG and FA was performed on a YMC DIOL column (4.6 × 250 mm, YMC, Asan). The liquid chromatography flow was divided, with approximately 80% directed to an FCII fraction collector (Waters, Milford, MA), and the remaining 20% directed to the evaporative light scattering detector (Waters, Milford, MA). Peak areas were quantified using Chemstation software (Agilent Technologies). Lipid analysis was performed at the Analytical Core for Metabolomics and Nutrition, British Columbia Children’s Hospital.

### 2.6 Quantitative real-time PCR and RNA sequencing

RNA from the frozen Vegfb^KO^ and WT control and diabetic heart ventricles was extracted via TRIzol (Invitrogen). Briefly, 20-50 mg frozen ventricle tissue powder was homogenized with 1 mL TRIzol reagent via glass homogenizer. Extracted RNA was washed with ethanol three times and reconstituted in DEPC water. Qualified mRNA was first reversely transcribed into cDNA, followed by cDNA amplification via TaqMan Fast Advanced Master Mix (ThermoFisher Scientific) and Taqman primer of targets (*Fatp4*: Rn01438951_m1, *Cd36*: Rn00580728_m1, *Vegfb*: Rn01454585_g1 and *B2m*: Rn00560865_m1) in StepOnePlus PCR instrument (Applied Biosystems). Gene expression was calculated based on comparative cycle threshold (ΔΔCT) with B2M as the reference gene. For each independent qPCR batch, the mean ΔCt of the WT group from the same batch was used as the calibrator and set to 1, and all samples from that batch were expressed relative to this WT reference.

The integrity of the RNA samples for sequencing was checked for quality by Bioanalyzer (Agilent Technologies) and quantified using a Qubit fluorometer (ThermoFisher Scientific). The sequencing library was prepared using NEBNext® Ultra II directional RNA library Prep Kit (Illumina) and sequenced using Illumina Nextseq500 (Illumina), collecting a total of 20 million paired reads. Multiple pipelines were applied to align to the rat reference transcriptome genome (Rattus_norvegicus.mRatBN7.2.112) and quantified for count reads at the transcript level for each gene for all samples as previously described (Vuilleumier et al., 2019; Puri et al., 2019). Differential expression analysis was performed with DESeq2 and edgeR packages in RStudio. No outliers were identified during sample clustering, so all samples were included in the differential expression analysis. The output from each pipeline was filtered for multiple tests with a cutoff: adjusted p-value less than 0.05 to ensure statistical significance. The filtered data list was then ranked by the adjusted p-value for differential expression. A combined output from all pipelines was then created by ranking the genes based on their median values. The differential expression was also determined by log2 fold change between control and diabetic rats within their own genotyping group. The secondary cut-off is an absolute log2 fold change larger than 0.27 to ensure biological significance. The network analysis and functional analysis were conducted via the clusterProfiler in RStudio with hypergeometric testing to calculate the adjusted p-values for each category. RNA sequencing activities were performed at the BRC Sequencing Core, University of British Columbia.

### 2.7 Western blot

Western blot analysis was performed as previously described (Lal et al., 2017). Protein extracted from the heart ventricle was visualized by the Li-COR Odyssey CLX digital imaging system, and bands were quantified by Image Studio™. Antibodies used in this project are listed: anti-α-Tubulin DM1A (Millipore, 05-829), anti-Vinculin (Cell Signaling Technology, 13901), anti-LPL 5D2 (Santa Cruz, sc-73646), and anti-Poly (ADP-ribose) polymerase (PARP) (Cell Signaling Technology, 9542).

### 2.8 Statistical analysis

All statistical analysis was generated via Graph Prism 10.0 (GraphPad Inc, San Diego, CA, United States). The Shapiro–Wilk test was performed to determine the normality of the data. For normalized data, unpaired Student’s t-test, or Two-way ANOVA followed by Bonferroni’s *post hoc* comparisons test was performed to determine the statistical significance. Otherwise, nonparametric analysis with the Mann-Whitney test or Wilcoxon test was performed. The results are presented as mean ± SEM with a *p* value <0.05, which was considered significant.

## 3 Results

### 3.1 Characterization of the global Vegfb^KO^ rat model

**FIGURE 1:**
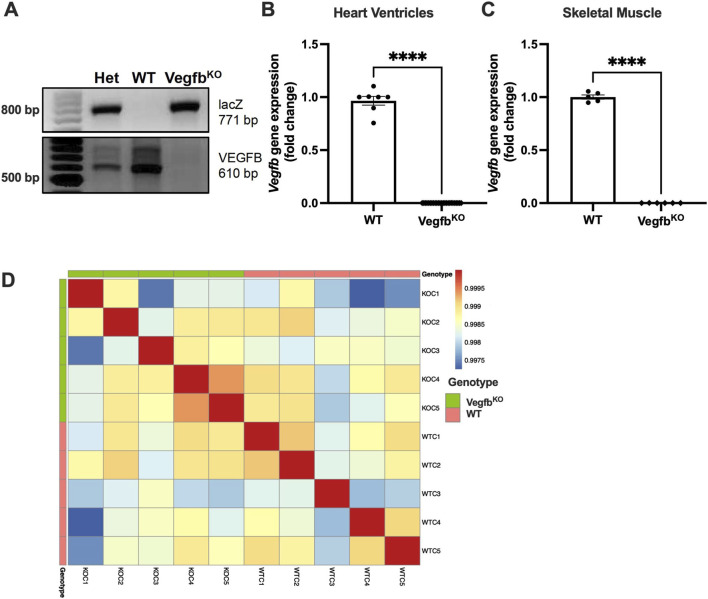
Characterization of the global Vegfb^KO^ rat model **(A)** PCR amplification of gDNA extracted from heterozygous, WT and Vegfb^KO^ rat ear notch samples, confirming Vegfb^KO^ via beta-galactosidase knock-in (Vegfb at 610 bp, LacZ at 771 bp). **(B,C)** mRNA expression of Vegfb in WT and Vegfb^KO^ rat heart ventricles (n = 7–25) and skeletal muscle (n = 5–6) as determined using quantitative RT-PCR, confirming Vegfb gene deletion. **(D)** Spearman’s correlation heatmap of transcriptomic analysis of WT and Vegfb^KO^ hearts (n = 5). Data are presented as mean ± SEM. Unpaired t-tests were used to compare the two groups, ****p < 0.0001.

The global Vegfb^KO^ rat was generated as previously described (Kivela et al., 2014) using zinc finger nucleases with beta-galactosidase knock-in to disrupt the *Vegfb* gene (Supplementary 1A). The gel electrophoresis illustrates the genotyping of gDNA extracted from heterozygous, WT and Vegfb^KO^ rat ear notch samples. Our results confirmed that the *Vegfb* gene was indeed deleted from Vegfb^KO^ rats (Figure 1A). As determined by quantitative RT-PCR, *Vegfb* gene expression was undetectable in the Vegfb^KO^ rat heart ventricles (Figure 1B), skeletal muscle (Figure 1C), liver and pancreas (data not shown) compared to the WT group, successfully validating the global knockout of *Vegfb* in the rat. To characterize the effect of Vegfb^KO^ at baseline conditions, we conducted RNA sequencing of the heart ventricles from WT and Vegfb^KO^ rats. Based on the principal component analysis, WT and Vegfb^KO^ rat points were intermixed with no clear separation, suggesting that Vegfb^KO^ had only a limited effect across the two groups. The gene expression of WT and Vegfb^KO^ groups was similar, and the differences might not be significant enough to be captured by the principal component analysis (Supplementary 1B). The Spearman correlation heatmap also showed similar results, suggesting that deletion of *Vegfb* under normal conditions did not cause a significant change in overall cardiac gene expression (Figure 1D).

### 3.2 Effect of Vegfb^KO^ on the severity of STZ-diabetes

**FIGURE 2:**
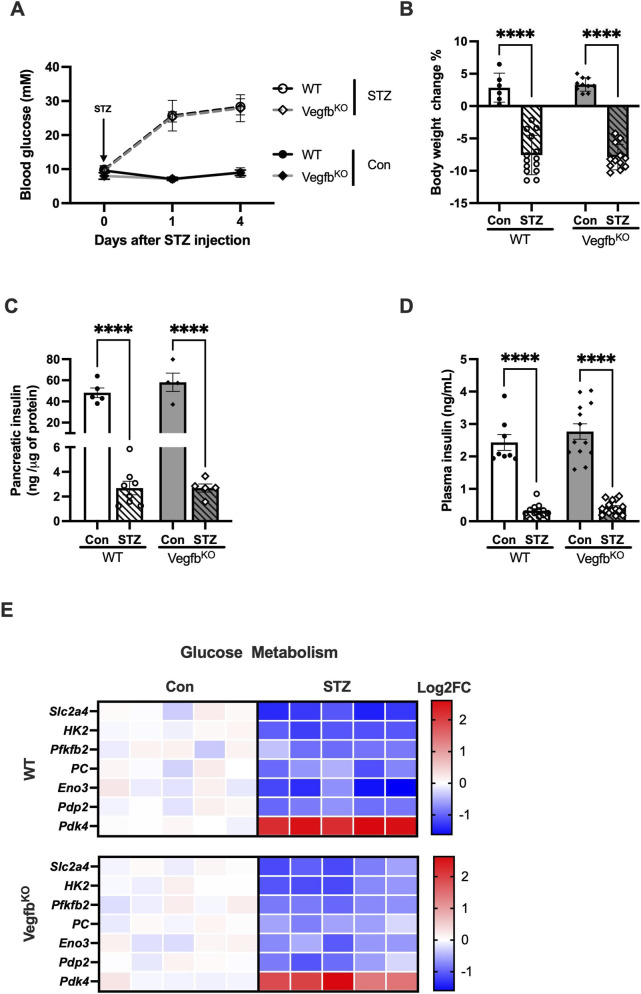
The severity of diabetes is similar in WT and Vegfb^KO^ rats injected with STZ **(A)** Blood glucose changes over the 4 days of STZ diabetes (n = 4–9). **(B)** Change in body weight in WT and Vegfb^KO^ rats after 4 days of STZ diabetes (n = 6–14). **(C)** Following acid-ethanol extraction, pancreatic insulin levels in the different groups were measured with an insulin ELISA (n = 4–8). **(D)** Plasma insulin in the different groups as measured (n = 8–15). **(E)** Key differentially expressed gene related to classic glucose metabolism genes as described using heat map (n = 5). Data are presented as mean ± SEM. Two-way ANOVA was used to analyze the data, ****p < 0.0001.

Injection of STZ successfully induced hyperglycemia in both WT and Vegfb^KO^ animals (Figure 2A), accompanied by a significant reduction in body weight over 4 days along with in these animals compared to their respective controls (Figure 2B). Moreover, diabetic WT rats exhibited markedly decreased pancreatic insulin levels (Figure 2C), which corresponded to the changes observed in plasma insulin (Figure 2D). Similar results were observed in the Vegfb^KO^ diabetic animals. Deletion of *Vegfb* did not have a significant influence on the overall characteristics of the rat, with no difference observed in glucose and FA metabolism under normal conditions. To assess whether this comparable level of diabetes was also evident in cardiac tissue, transcriptome analysis was performed. Hearts from both diabetic groups exhibited an almost similar reduction in the expression of genes related to glucose metabolism compared to their respective controls, suggesting a comparable impairment in cardiac glucose utilization. However, Vegfb^KO^ diabetic animals appeared to still demonstrate better glucose metabolism compared to WT diabetic. These data suggest that although WT and Vegfb^KO^ rats are equally susceptible to STZ diabetes based on general characteristics, *Vegfb* knockout lessens the dysfunction in cardiac glucose utilization following diabetes (Figure 2E).

### 3.3 Cardiac heparin-releasable LPL activity in Vegfb^KO^ rats following diabetes

**FIGURE 3:**
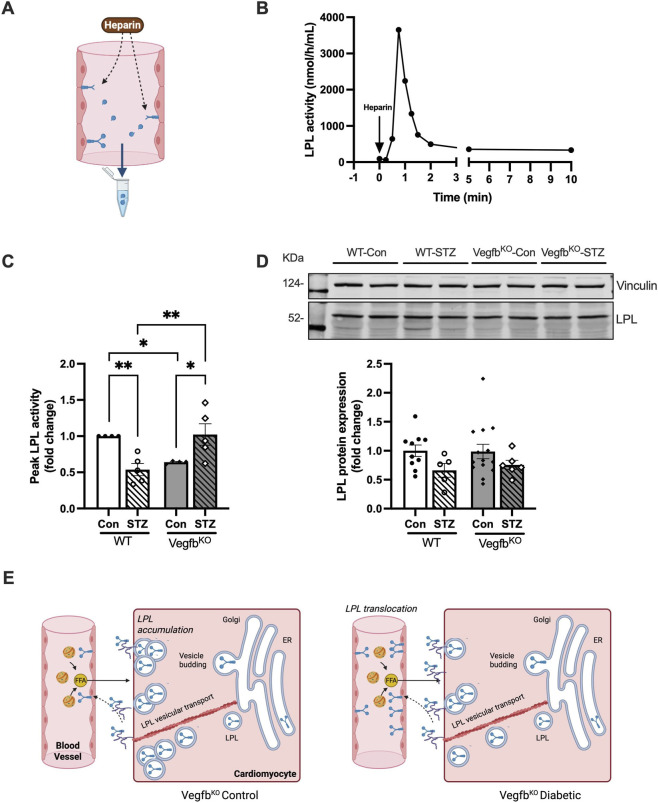
Vegfb^KO^ increases coronary vascular LPL activity following diabetes **(A)** Vascular LPL activity was determined by perfusing the isolated Langendorff heart with the highly negatively charged heparin and collecting the coronary perfusate over time for the determination of LPL activity. **(B)** Representative example of heparin-releasable LPL activity from a WT control heart following 10 min of heparin (5 U/mL; arrow) perfusion. Fractions of perfusate (over 15 s) were collected at the indicated times and analyzed for LPL activity. **(C)** Peak heparin-releasable LPL activity from hearts from the different groups was normalized to the peak LPL from WT control rats. Only the fold change is presented (n = 4–5). **(D)** Western blot of LPL protein from intact hearts isolated from different groups of rats (n = 5–14). The inset is a representative example of WT and Vegfb^KO^ heart samples following diabetes. **(E)** Potential mechanism by which Vegfb knockout affects coronary vascular LPL. In Vegfb^KO^ hearts, LPL is prevented from translocating to the vascular lumen and possibly accumulates in the cardiomyocytes (left panel); this effect is reversed with diabetes (right panel). Two-way ANOVA was used to analyze the data. Data are presented as mean ± SEM, *p < 0.05, **p < 0.005.

LPL is an important enzyme that regulates FA delivery to the (An and Rodrigues, 2006). To address the impact of Vegfb^KO^ on cardiac LPL activity, isolated hearts were retrogradely perfused with heparin to displace LPL bound to the luminal surface of coronary EC binding sites. LPL released into the perfusion buffer was collected, and its activity subsequently measured (Figure 3A). Addition of heparin induced a rapid increase in LPL activity in the coronary perfusate, with peak LPL activity appearing within the first minute and quickly returning to basal levels within 2 minutes (Figure 3B). Interestingly, hearts from Vegfb^KO^ control animals exhibited a significant reduction in peak LPL activity compared with WT controls, indicating decreased LPL activity at the vascular lumen of Vegfb^KO^ hearts (Figure 3C). This reduction in enzyme activity occurred in the absence of any change in LPL protein expression in the heart (Figure 3D), suggesting that VEGFB deficiency may impair the translocation of LPL from the cardiomyocyte to the vascular lumen (Figure 3E, left panel). In the heart, insulin plays an important role in regulating LPL. In severe diabetes, the reduction in insulin is known to decrease coronary LPL activity (Shang and Rodrigues, 2021). As anticipated, WT diabetic hearts had a significant reduction in peak LPL activity compared to their controls. Intriguingly, a marked increase in peak LPL activity was observed in Vegfb^KO^ hearts following diabetes, compared to their own controls and the WT diabetic group (Figure 3C). Despite these alterations in cardiac LPL activity, LPL protein levels remained unchanged across all four groups (Figure 3D). Our data suggest that in Vegfb^KO^ animals, the lower LPL activity in this group maybe a consequence of LPL protein accumulation inside the cardiomyocyte with limited movement to the EC surface. Following diabetes and its reduced utilization of glucose, this accumulated LPL starts to move to the EC lumen (Figure 3E, right panel), such that in Vegfb^KO^ diabetic hearts, LPL is likely a major mechanism for FA delivery.

### 3.4 Effect of Vegfb^KO^ on the plasma and cardiac lipid profile after diabetes

**FIGURE 4:**
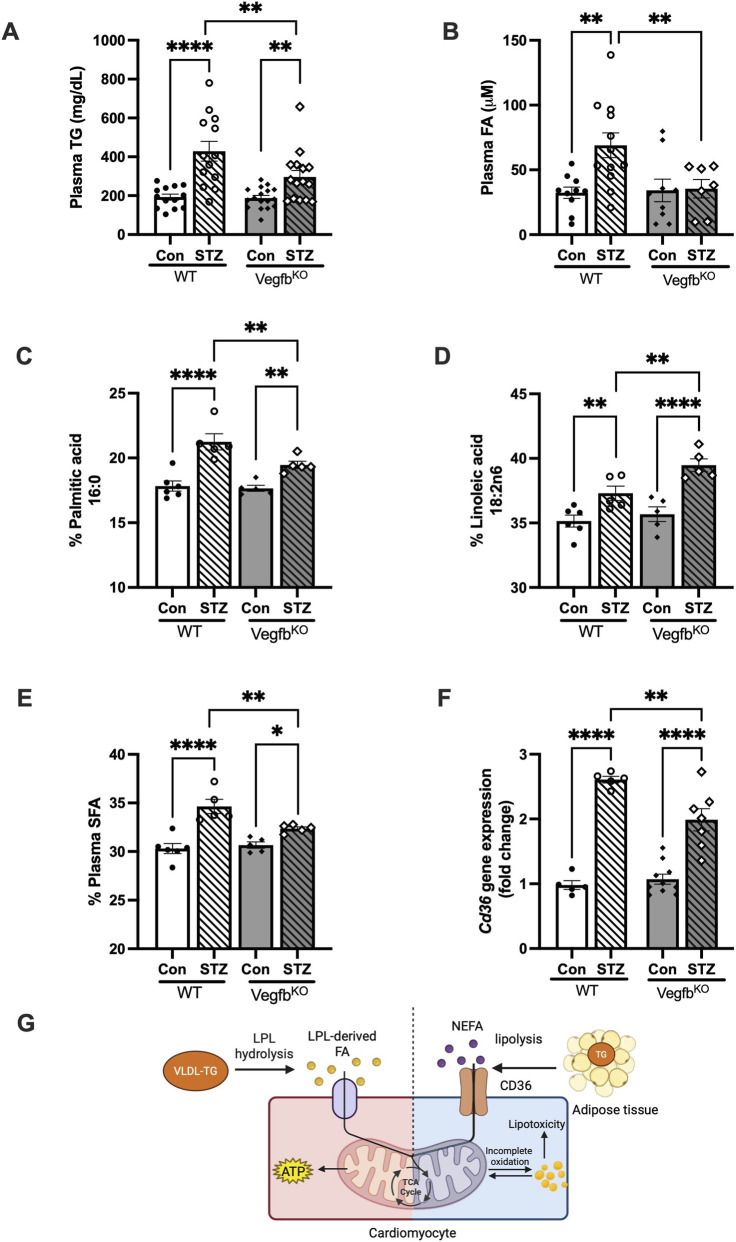
Plasma lipid profile in WT and Vegfb^KO^ control and diabetic animals **(A)** Plasma triglycerides during diabetes (n = 13–17). **(B)** Total plasma FA levels following diabetes (n = 7–12). **(C)** Percentage of palmitic acid in the plasma (n = 5–6). **(D)** Percentage of linoleic acid in the plasma (n = 5–6). **(E)** Percentage of saturated FA (SFA) in the plasma (n = 5–6). **(F)** Expression of the FA transporter, Cd36 in the heart (n = 5–10). **(G)** Mechanism by which NEFA and LPL-derived FA contribute towards TG storage or oxidation. Vegfb^KO^ hearts use more LPL-derived FA as opposed NEFA, which in WT diabetes saturates mitochondria oxidation, leading to lipotoxicity. Data are presented as mean ± SEM. Two-way ANOVA was used to analyze the data, *p < 0.05,**p < 0.005, ****p < 0.0001.

As cardiac LPL plays a significant role in clearing plasma TG (An and Rodrigues, 2006), we investigated the relationship between LPL activity and circulating lipids in Vegfb^KO^ control and diabetic rats. Under normal conditions, plasma TG levels were comparable between WT and Vegfb^KO^ groups, despite the lower cardiac LPL activity in Vegfb^KO^ hearts. Following diabetes, reduced cardiac LPL in WT diabetic rats was associated with elevated plasma TG, whereas higher LPL activity in Vegfb^KO^ diabetic hearts corresponded to a less severe hypertriglyceridemia (Figure 4A). These findings indicate that deletion of *Vegfb* had beneficial effects on plasma TG clearance. In addition, induction of diabetes caused a significant increase in total plasma FA in the WT diabetic groups. Intriguingly, global VEGFB deficiency protected against this diabetes-induced elevation in circulating total FA levels (Figure 4B).

Subsequently, we assessed the proportional changes in individual FA species between the two diabetic groups (Table 1). After 4 days of hyperglycemia and hypoinsulinemia, plasma levels of both palmitic and linoleic acids were elevated. Notably, Vegfb^KO^ diabetic animals exhibited lower palmitic acid and higher linoleic acid levels compared with WT diabetic groups. (Figures 4C,D). This difference in plasma FA change was more evident when the overall plasma FA composition was evaluated as the saturated FA (SFA) fraction. Compared to Vegfb^KO^ diabetic rats, the plasma SFA was significantly higher in WT diabetic rats (Figure 4E).

**TABLE 1:** Plasma fatty acid percentages (mean ± SEM).

Key fatty acids	Wildtype		Vegfb^KO^	
	Con	STZ	Con	STZ
Palmitic acid	17.83 ± 0.39	21.24 ± 0.62	17.66 ± 0.23	19.46 ± 0.28
Stearic acid	6.83 ± 0.31	8.64 ± 0.42	7.68 ± 0.42	8.44 ± 0.39
Oleic acid	11.48 ± 0.51	9.74 ± 1.01	11.04 ± 0.50	10.22 ± 0.17
Linoleic acid	35.15 ± 0.46	37.3 ± 0.56	35.68 ± 0.57	39.48 ± 0.49
Arachidonic acid	10.1 ± 0.66	9.32 ± 1.12	10.56 ± 0.87	8.66 ± 0.65

LPL-derived FA and NEFA enter cardiomyocytes using different mechanisms, one being transporter mediated (van der Vusse et al., 2000). We measured the major FA transporters *Fatp4* and *Cd36* expression in hearts from the different groups. Although there was no difference observed in cardiac *Fatp4* levels (Supplementary 1C), Vegfb^KO^ diabetic hearts had significantly lower *Cd36* expression compared with WT diabetes (Figure 4F). As CD36 is a major transporter of NEFA (Coburn et al., 2000; Hwang et al., 1998; Bharadwaj et al., 2010; He et al., 2018), our data suggest that hearts from WT diabetic animals use more NEFA due to decreased LPL activity, a potential mechanism that could lead to unregulated FA delivery and subsequent lipotoxicity (Figure 4G).

### 3.5 Outcome of Vegfb^KO^ on the transcriptome especially related to cardiac lipid metabolism during diabetes

**FIGURE 5:**
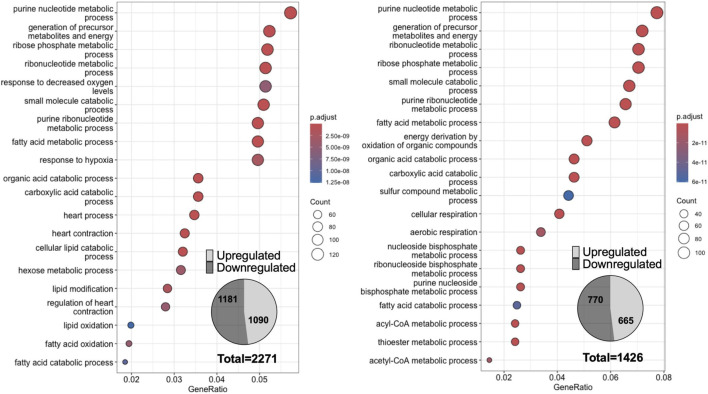
Dotplots of the ventricular transcriptome of rats with diabetes clustered based on biological process. Dot plots display the top 20 most enriched GO biological process terms by gene ratio (the number of genes related to GO term/total number of significant genes). The adjusted *p* value is represented by color, and the size of the circle represents the counts involved in each biological process. Left panel is WT control versus WT diabetic rats. Right panel is Vegfb^KO^ control versus Vegfb^KO^ diabetic rats. The insets describe the total number of genes whose expression was either up- or downregulated in each comparison (n = 5).

**FIGURE 6:**
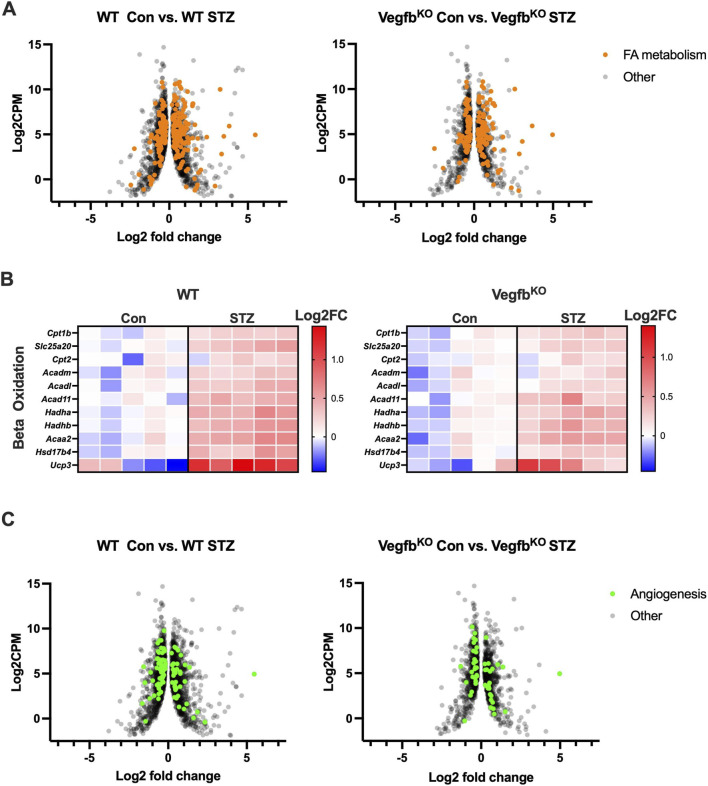
Ventricular transcriptome with emphasis on FA metabolism and angiogenesis in rats with diabetes **(A)** The volcano plots describe the profile of differentially expressed genes associated with FA metabolism in WT and Vegfb^KO^ rats following diabetes (n = 5). **(B)** The heat map was used to represent the key differentially expressed gene related to beta oxidation (n = 5). **(C)** Volcano plot expresses differentially expressed genes linked with angiogenesis in WT and Vegfb^KO^ rats following diabetes (n = 5). The x-axis represents Log2FC expression of genes versus Log2CPM on the y-axis. CPM indicates counts per million.

To evaluate overall cardiac FA metabolism, heart ventricle transcriptome in WT and Vegfb^KO^ groups following diabetes were compared. Two cut-offs (adjusted p-value less than 0.05 and gene fold change larger than 1.2) were set to filter genes that were both statistically significant and biologically meaningful, improving the robustness and interpretability of our results. Interestingly, WT diabetic animals exhibited dramatic transcriptomic changes with 2271 differentially expressed genes when diabetes was induced. However, Vegfb^KO^ diabetic animals showed less severe transcriptomic changes with 1426 differentially expressed genes (Figure 5). Clustering analysis revealed that the top 20 most significantly altered biological processes in both WT and Vegfb^KO^ groups under diabetic conditions were primarily related to lipid and nucleotide metabolism. (Figure 5). When looking specifically into FA metabolism, changes in genes were more pronounced (234 genes) in the WT compared with the Vegfb^KO^ group after diabetes (172 genes) (Figure 6A), with more genes related to TG breakdown and FA oxidation being upregulated, likely a response to a greater oxidation of NEFA (Figure 6B). Furthermore, additional changes were observed in the top 20 most significantly altered biological processes associated with cardiac function and hypoxia in the WT diabetic hearts. Unlike glucose, FA oxidation requires more oxygen delivered via blood vessels (Kessler and Friedman, 1998). It has been reported that diabetes will cause microangiopathy (SAPRA et al., 2024). Although both diabetic animals showed significant changes in genes controlling angiogenesis, this was more apparent in the WT diabetic group (WT 100 genes vs. Vegfb^KO^ 52 genes) (Figure 6C). Our data suggest that with an excess of FA metabolism and inadequate oxygen supply in the WT diabetic group, there is a greater susceptibility for lipotoxicity in this group compared to Vegfb^KO^ diabetic rats.

### 3.6 Effect of Vegfb knockout on cardiac cell death after diabetes

**FIGURE 7:**
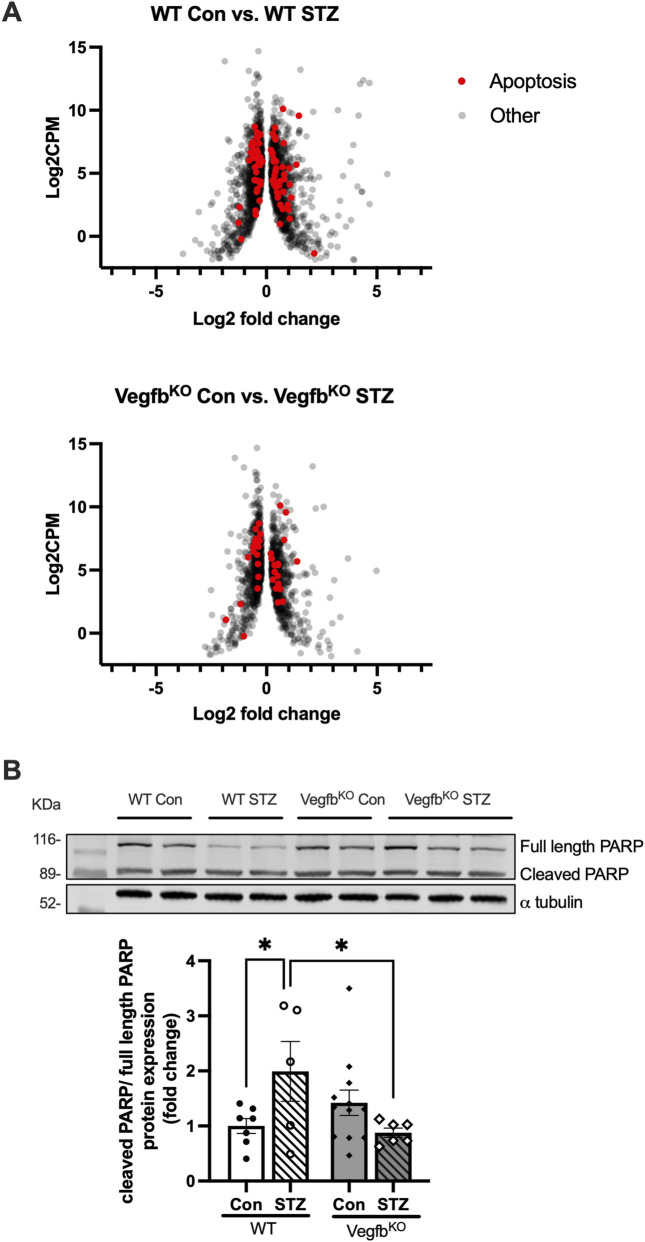
Ventricular transcriptome and protein expression related to cell death in rats with diabetes **(A)** The volcano plots describe the profile of differentially expressed genes associated with cell death in WT and Vegfb^KO^ following diabetes (n = 5). The x-axis represents Log2FC expression of genes versus Log2CPM on the y-axis. CPM indicates counts per million. **(B)** The expression of cleaved PARP/full length PARP in hearts from the different groups (n = 5–13). The inset is a Western blot representing WT and Vegfb^KO^ hearts following diabetes. Two-way ANOVA was used to analyze the data. Data are presented as mean ± SEM, *p < 0.05.

Increased plasma SFA, oxidative stress and lipotoxicity due to impaired angiogenesis have all been linked to ER stress, ROS generation and cell death (Laakso, 2011; Hinkel et al., 2017; Adameova and Dhalla, 2014; Palomer et al., 2018; Blanchette-Mackie et al., 1989; Shang et al., 2021; Borradaile and Schaffer, 2005; Wilson et al., 2018; Volpe et al., 2018; van de Weijer et al., 2011). Indeed, there were more genes related to apoptosis that were altered in the WT group compared to the Vegfb^KO^ group during diabetes (WT 80 genes vs. Vegfb^KO^ 36 genes) (Figure 7A). Taken together with a decrease in cardiac cleaved PARP levels in Vegfb^KO^ diabetic rats compared to WT diabetic rats (Figure 7B), our results suggest that Vegfb^KO^ may protect the heart from cell death during diabetes.

## 4 Discussion

Following diabetes, the heart has a limited capacity to utilize glucose for energy and thus relies on FA. Regrettably, LPL-derived FA, a major source, is turned off during severe diabetes (Willecke et al., 2015; Puri et al., 2019) and the heart shifts to predominantly using NEFA. This shift in cardiac metabolism contributes to the development of DbCM, one cause of death in patients with diabetes. Vegfb^KO^ in mice is known to decrease white adipose tissue (WAT) lipolysis following a high fat diet (HFD) (Falkevall et al., 2023). We tested whether Vegfb^KO^ in rats could be potentially beneficial in a T1D model of diabetes. Our data suggests that Vegfb^KO^ in STZ diabetes has a protective role in alleviating the plasma lipid profile through its action on LPL, improves metabolic flexibility by enhancing glucose utilization, and augmenting cardiac cell survival.

Rats with global depletion of *Vegfb* gene exhibit no significant transcriptomic changes in the heart, or any change in body weight, plasma lipids or insulin levels. This was similar to *Vegfb* deficiency mice (Falkevall et al., 2023; Falkevall et al., 2017; Ling et al., 2022; Dijkstra et al., 2014; Cao et al., 2023; Hagberg et al., 2012). Unlike Vegfb^KO^, cardiac-specific overexpression of *Vegfb* induces a remarkable transcriptomic change with over 2000 genes significantly differentially expressed (Shang et al., 2021). Whether these changes are due to a direct or indirect effect of *Vegfb* overexpression is currently unknown. However, it should be noted that in these *Vegfb* transgenic rats, there was a significant increase in coronary blood vessels with enhanced insulin signaling, suggesting that this later effect may have contributed towards alterations in the metabolic transcriptome (Shang et al., 2024; Robciuc et al., 2016). Although no apparent changes were observed in rats or mice following Vegfb^KO^, when Vegfb^KO^ mice were exposed to a stress like HFD or constructed onto the *db/db* model, a significant improvement was observed in plasma lipids, tissue fat accumulation, insulin sensitivity, pancreatic insulin expression, and glucose and insulin tolerance suggesting that *Vegfb* knockout does have an beneficial impact in the context of environmental stressors like T2D (Falkevall et al., 2023; Falkevall et al., 2017; Ling et al., 2022; Dijkstra et al., 2014; Cao et al., 2023; Hagberg et al., 2012; Ning et al., 2020). In our study, although there were no significant differences observed in the general characteristics between Vegfb^KO^ and WT rats in response to STZ diabetes, transcriptome analysis suggested that the Vegfb^KO^ diabetic group appeared to exhibit improved cardiac glucose metabolism compared to WT diabetes. Related to glucose uptake, Hagberg’s group has shown that Vegfb^KO^ in mice enhanced cardiac glucose uptake by regulating GLUT4 levels (Hagberg et al., 2010), and treating mice with VEGFB neutralizing antibodies improved glucose uptake to the heart with increased cardiac glycogen (Moessinger et al., 2020). Conversely, VEGFB treatment inhibited endothelial glucose transcytosis by decreasing GLUT1 levels. Overall, these results suggest that inhibition of VEGFB can improve glucose utilization in a T1D model, similar to a model of T2D.

In the heart, LPL is functional at vascular lumen where it breaks down circulating TG to FA. Under control conditions, luminal LPL activity was significantly lower without any change in the total LPL protein in Vegfb^KO^ rat hearts, suggesting that VEGFB contributes towards LPL translocation from the cardiomyocyte to the EC. In support, incubating primary isolated cardiomyocytes with recombinant VEGFB increased cardiomyocyte surface LPL activity by activating p38 mitogen-activated protein kinase (MAPK) signaling (Shang et al., 2024). It has been known that phosphorylation of p38 MAPK can initiate F-actin cytoskeleton polymerization to increase cardiomyocyte surface LPL activity (Kim et al., 2008a). Our data for the first time suggests that *Vegfb* knockout impedes LPL translocation from the cardiomyocyte to the EC likely resulting in its accumulation in the cardiomyocyte. Inexplicably, rats with cardiac specific overexpression of *Vegfb* also exhibited reduced luminal LPL activity with associated accumulation of LPL within cardiomyocytes (Shang et al., 2024). Nevertheless, we proposed that in these *Vegfb* transgenic animals, augmented angiogenesis and increased insulin delivery were responsible for these effects, with enhanced insulin action reprograming the heart to favor glucose utilization, instead of using LPL-derived FA (Shang et al., 2021; Robciuc et al., 2016). In diabetes, increasing circulating FAs has been reported to inhibit LPL activity (Yu et al., 2005; Wang et al., 2011; Kristensen et al., 2020; Wang et al., 2009; Kim et al., 2009). Consistent with previous findings, elevated plasma NEFA following STZ-diabetes induction diminished LPL activity in WT rats. Interestingly, enhanced LPL activity was observed in Vegfb^KO^ diabetic hearts, with no difference in total LPL protein among the four groups. These results suggest that the increased LPL activity in the Vegfb^KO^ diabetic group is likely a result of the accumulated LPL in cardiomyocytes being translocated to the coronary vessels following insulin reduction.

Cardiac LPL activity is important for regulating plasma TG (An and Rodrigues, 2006). With decreased LPL activity in Vegfb^KO^ rats under normal conditions, we expected lower TG clearance and higher plasma TG in these animals. However, comparable plasma TG was observed between the WT and Vegfb^KO^ groups suggesting that other factors including reduced hepatic TG secretion may have contributed to this effect (Willecke et al., 2015). In this regard, as FA derived from WAT contributes significantly to hepatic TG synthesis (Schweiger et al., 2014; Alves-Bezerra and Cohen, 2017), it is possible that a reduction in WAT lipolysis could influence hepatic FA uptake and TG synthesis. Indeed, inhibition of VEGFB promotes lipid storage in WAT under normal conditions (Hagberg et al., 2010). Given the association between cardiac LPL activity and plasma TG (Willecke et al., 2015; Levak-Frank et al., 1999), it was not surprising that the WT diabetic rats exhibited hypertriglyceridemia. Intriguingly, Vegfb^KO^ diabetic rats exhibited less severe hypertriglyceridemia, which could be explained by the increase in LPL activity. A correlation between hypertriglyceridemia and C-reactive protein levels (an inflammation marker) has been reported in humans (Kraaijenhof and Stroes, 2023). In addition to hypertriglyceridemia, diabetes is also responsible for an inflammatory response. An increase in inflammation markers, upregulation of pro-inflammatory genes, and pathological angiogenesis have been observed in adipocyte-specific *Vegfb* overexpressing mice under HFD conditions, effects that were not observed in *Vegfb* deficient mice (Falkevall et al., 2023). In parallel, administering anti-VEGFR1 antibodies was found to decrease the incidence of arthritis, synovial inflammation and atherosclerosis in mice. Notably, the suppression of VEGFR2 did not yield an anti-inflammatory effect in mice with arthritis or atherosclerosis (Luttun et al., 2002), implying that this inflammation effect is probably related to VEGFB-VEGFR1 signaling. Given that Vegfb^KO^ diabetic rats have relatively normal plasma TG together with the known anti-inflammatory effects associated with *Vegfb* knockout (Falkevall et al., 2023; Luttun et al., 2002), our data imply that suppression of VEGFB action protects against hypertriglyceridemia by augmenting cardiac LPL and this may be beneficial in reducing inflammation and its related diseases like diabetes.

Under normal conditions, total plasma FA profiles were indistinguishable between WT and Vegfb^KO^ groups. However, induction of diabetes caused a significant increase in total plasma FA in WT rats, whereas Vegfb^KO^ diabetic rats maintained normal plasma FA levels. One potential mechanism could be a decreased WAT lipolysis in Vegfb^KO^ diabetic rats. Indeed, adipocyte-specific *Vegfb*-deficient mice display reduced WAT lipolysis and relatively normal total plasma FA under HFD (Falkevall et al., 2023). During diabetes, WT rats demonstrated significantly higher palmitic acid levels and lower linoleic acid percentage compared to Vegfb^KO^ diabetic rats. Palmitate acid, a typical SFA, is known to have deleterious effects on cardiac function (Yamamoto and Sano, 2022). Palmitic acid can bind to toll-like receptor 4, triggering pro-inflammatory gene expression and contributing to insulin resistance (Wang et al., 2017). Moreover, treating cardiomyocytes with palmitate acid induces palmitoylation of CD36 (Zhang et al., 2025), which allows CD36 translocation to the cell membrane with subsequent FA uptake (Luse et al., 2025; Zhang et al., 2025). Preventing CD36 palmitoylation protects the heart from lipid overload, mitochondria dysfunction, lipotoxicity (Luse et al., 2025; Zhang et al., 2025) and enhances autophagy (Zhang et al., 2025). Additionally, mice fed a diet rich in SFA have elevated SFA composition in both plasma and ER membranes in the heart, causing membrane stiffness, ER stress and left ventricle diastolic dysfunction (Yamamoto et al., 2019). In contrast, increasing intake of linoleic acid, a polyunsaturated FA, is linked to a decreased risk of cardiovascular disease (Farvid et al., 2014). Our data imply that *Vegfb* knockout protects the heart against dysfunction during diabetes by decreasing total plasma FA, reducing toxic palmitate acid and increasing beneficial lipids like linoleic acid.

VEGFB inhibition has also been reported to decrease FA uptake and lipid accumulation in non-adipose tissues (Hagberg et al., 2010; Falkevall et al., 2023; Falkevall et al., 2017). Deletion of *Vegfb* in both rat and mice decreased the FA transporter *Fatp4* levels in the heart under control conditions (Hagberg et al., 2010; Kivela et al., 2014). However, we did not observe any change in either FA transporters (*Fatp4* and *Cd36*) or key cardiac FAs in rat ventricular muscle, implying that global *Vegfb* knockout in rats did not influence FA uptake in the heart under normal conditions. Without an effect on FA uptake, *Vegfb* knockout is unlikely to impact cardiac lipid content. Indeed, no difference in cardiac TG and FA was observed between WT and Vegfb^KO^ rats (Supplementary Figure S1 and Table 2). In accordance with our results, Alitalo’s group also found no difference in FA uptake and similar cardiac TG levels in both WT and Vegfb^KO^ rats (Kivela et al., 2014).

**TABLE 2:** Cardiac fatty acid percentages (mean ± SEM).

Key fatty acids	Wildtype		Vegfb^KO^	
	Con	STZ	Con	STZ
Palmitic acid	11.22 ± 0.23	11.1 ± 0.36	10.64 ± 0.12	10.62 ± 0.47
Stearic acid	19.38 ± 0.24	21.26 ± 0.30	19.88 ± 0.16	20.86 ± 0.47
Oleic acid	4.47 ± 0.17	6.98 ± 1.06	4.28 ± 0.24	5.96 ± 1.40
Linoleic acid	27.10 ± 0.43	23.78 ± 0.69	26.54 ± 0.49	23.66 ± 1.01
Arachidonic acid	14.53 ± 0.42	13.88 ± 0.80	14.56 ± 0.12	15.34 ± 1.11

Based on the FA sources, external FA can be classified as LPL-derived FA or NEFA. Both plasma FA and LPL-derived FA need to be taken up mainly through transporters, with CD36 being critical for NEFA uptake (Coburn et al., 2000; Hwang et al., 1998; Bharadwaj et al., 2010; He et al., 2018). Indeed, when radioactive FA was injected into both WT and CD36 null mice, *Cd36*-deficient mice had a significant decrease in FA uptake into multiple tissues including the heart (Coburn et al., 2000). Similar results were also observed in healthy and *CD36*-deficient humans (Hwang et al., 1998). However, although knockout of *Cd36* in mice decreased NEFA uptake, it did not impact the uptake rate of LPL-derived FA into cardiomyocytes following the injection of radiolabelled FA and VLDL (He et al., 2018). Also, CD36 is not necessary for the uptake of FA derived from chylomicron lipoproteins, as the high FA concentration generated from chylomicron-TG lipolysis facilitates the majority of FA uptake through passive diffusion (Bharadwaj et al., 2010). These results imply that CD36 is unlikely to be responsible for the uptake of LPL-derived FA. With relatively lower CD36 expression and elevated LPL activity in the heart compared to WT diabetic groups, Vegfb^KO^ diabetic animals may rely less on NEFA, and more on LPL-derived FA, which is considered a better FA source for the following reasons. Cardiomyocytes can regulate LPL translocation by modulating LPL-derived FA delivery through the AMPK pathway in response to energy demand (An et al., 2005; Kim et al., 2008b). Furthermore, our lab showed that the NEFA uptake rate was almost equivalent to the NEFA oxidation rate in the fasting rat hearts (Shang et al., 2024). These findings imply that NEFA is preferentially oxidized following uptake, which may contribute to oxidative stress (Shang et al., 2024). Moreover, lipoproteins contain substantial FA content, thus offering a richer FA supply than other sources for ATP generation (Chiu et al., 2016). We conclude that *Vegfb* knockout in diabetes contributes towards the use of a better FA source, LPL-derived FA, compared to NEFA.

Following uptake, NEFA are preferentially oxidized (Shang et al., 2024). Given that WT diabetic animals had excess NEFA uptake into the heart, it was not surprising that these hearts had a greater number of genes differentially expressed that were associated with FA metabolism compared to the Vegfb^KO^ groups, implying a higher oxidative stress in WT diabetes. As this WT diabetic group also had more genes related to angiogenesis and hypoxia response that were regulated, it is likely that incomplete lipid oxidation causes the accumulation of toxic lipid intermediates (Chou et al., 2002; Kessler and Friedman, 1998; Hinkel et al., 2017). Indeed, given that more genes related to cell death were differentially expressed and more protein cell death markers were detected in the WT diabetic animals than in the Vegfb^KO^ group, it suggests that inhibition of Vegfb has a protective effect against diabetes induced cell death. This finding is consistent with the pro-survival effect of *Vegfb* inhibition on pancreatic tumors (Albrecht et al., 2010).

During diabetes, increased FA utilization for energy generation due to limited capacity in glucose utilization causes oxidative stress and lipid accumulation in the heart, which contributes significantly to the development of diabetic DbCM. Vegfb^KO^ had minimal phenotype and transcriptome changes under normal conditions. However, following diabetes, Knockout of *Vegfb* in rats is beneficial to mitigate cell death through *i)* alleviation of hyperlipidemia by upregulating cardiac LPL activity and *ii)* prevention of excess FA oxidation by providing more LPL-derived FA to the heart. Inhibiting VEGFB may offer a promising therapeutic approach to protect the heart from diabetes-induced cardiomyopathy. Further investigation of the mechanism behind how VEGFB inhibition modulates LPL translocation could provide valuable insights for drug development and help fill the gap in mechanism-based treatments for DbCM.

## Data Availability

The data presented in the study are deposited in the GEO repository, accession number GSE318258.

## Supplementary material

SUPPLEMENTARY FIGURE S1
**(A)** Process of generating a systemic *Vegfb* gene knockout rat by zinc finger nucleases with beta-galactosidase knock-in. **(B)** Principal component analysis of transcriptomic analysis of WT and Vegfb^KO^ hearts (n=5). **(C)** Expression of *Fatp4* in the heart (n=5-12). **(D)** Cardiac TG content (n=5-6). **(E)** The percentage of individual FAs that make up cardiac TG (n=5-6). Data are presented as mean ± SEM. Two-way ANOVA was used to analyze the data, * *p* < 0.05.
