# Supplementary figures and images for: Global knockout of VEGFB improves lipoprotein lipase activity leading to an improved lipid profile during diabetes
Source: Front Pharmacol. 2026 Feb 18;17:1759414. doi: 10.3389/fphar.2026.1759414 (PMC12957161; doi:10.3389/fphar.2026.1759414)

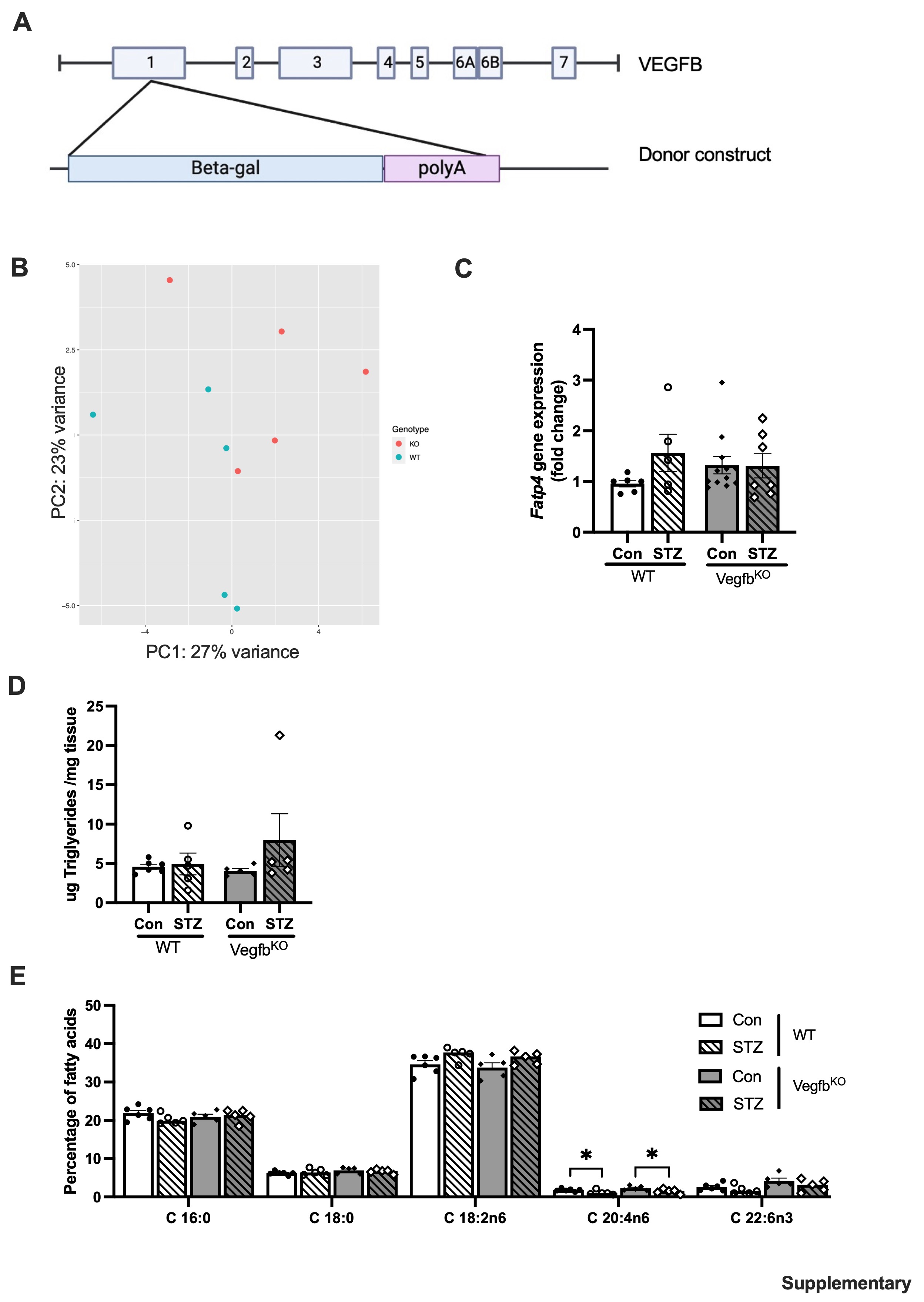

Supplement: Supplementary file 1 [file Image1.jpeg]
